# Year-round individual specialization in the feeding ecology of a long-lived seabird

**DOI:** 10.1038/s41598-019-48214-0

**Published:** 2019-08-14

**Authors:** Laura Zango, José Manuel Reyes-González, Teresa Militão, Zuzana Zajková, Eduardo Álvarez-Alonso, Raül Ramos, Jacob González-Solís

**Affiliations:** 1 Institut de Recerca de la Biodiversitat (IRBio) and Department de Biologia Evolutiva, Ecologia i Ciències Ambientals (BEECA), Universitat de Barcelona, Av Diagonal 643, Barcelona, 08028 Spain; 2 Centre d’Estudis Avançats de Blanes (CEAB-CSIC), Blanes, 17300 Spain

**Keywords:** Behavioural ecology, Stable isotope analysis

## Abstract

Many generalist species are composed of individuals varying in the size of their realized niches within a population. To understand the underlying causes and implications of this phenomenon, repeated samplings on the same individuals subjected to different environmental conditions are needed. Here, we studied individual specialization of feeding strategies in breeding and non-breeding grounds of Cory’s shearwaters (*Calonectris borealis*) for 2–8 years, and its relationship with fitness. Individuals were relatively flexible in non-breeding destinations, but specialized in diet, habitat use and daily activity across years. Daily activity was also consistent throughout the year for the same individual, suggesting that it is driven by individual constraints, whereas individual diet and habitat use changed between breeding and non-breeding grounds, indicating that these specializations may be learned at each area. Moreover, individuals that were intermediate specialized in their diet tended to show higher breeding success than those with weakly and highly specialized diets, suggesting stabilizing selection. Overall, this study suggests that the development of individual specialization is more flexible than previously thought, i.e. it emerges under specific environmental conditions and can develop differently when environmental conditions vary. However, once established, individual specialization may compromise the ability of individuals to cope with environmental stochasticity.

## Introduction

In ecology, it has been traditionally assumed that individuals of the same population are ecologically equivalent^1^. However, populations of many apparently generalist species are in fact composed of individuals with different degrees of specialization, varying in their realized niches within a population^2,3^. This means that individuals consistently exploit a subset of resources, although resources are potentially available for all individuals in the population. These differences may not be attributable to specific classes, such as sex, age or morphotypes, but to differences among individuals, known as individual specialization^4^. Although initially referred to as trophic specialization, individuals have been reported to specialize in different habitats^5^, in more nocturnal/diurnal behaviour or in migration and foraging patterns in terms of timing, routes or areas exploited^6–8^.

The fact that individuals of the same species consistently differ in their biological traits has broad implications in ecology, evolution and conservation^4,9^. Ecologically, individual differences in biological traits may have broad consequences on individual fitness. Individual differences in habitat use or diet can influence breeding success. For example, neritic foragers showed better reproductive performance than oceanic ones in the loggerhead sea turtle (*Caretta caretta*)^10^, whereas western gulls (*Larus occidentalis*) specialized in exploiting human waste showed significantly lower long-term reproductive success than those specialized in fish^11^. Individual specialization is also key in evolutionary processes, since differences in resource exploitation among individuals may reduce intraspecific competition and promote ecological segregation^12^. Moreover, long-term individual specialization may limit behavioural plasticity and thus responses to changes in the environment^13^. That is, responses to a changing environment may occur within a generation if individuals are plastic or flexible in their strategies, whereas in populations composed of a majority of highly specialized individuals, adaptation will occur via natural selection^4^. From a conservation point of view and in the context of anthropogenic changes, behavioural plasticity at both the individual and population levels is therefore key because adaptation via natural selection is normally too slow for responding to rapid and extensive human-induced changes^14^.

Despite its implications, few studies have focussed on understanding the degree of individual specialization in several dimensions of the ecological niche simultaneously, precluding more general conclusions about its population consequences. This may be due to the difficulty of gathering long-term time series of individual longitudinal data^15^, which is often complicated for wild species. Although individual specialization has been broadly studied in past decades, many studies on wild-living animals are short-term, only including observations within the same season or for up to 2–4 years^4,6,7,16–18^ (but see some exceptions^19,20^). Although there is no consensus on the length of the period needed to study individual specialization, short-term studies may not capture the entire individual repertoire of the traits considered^21^, leading to a gross overestimation of individual specialization.

Little is known about the processes underlying individual specialization. Differences among individuals in resource use reflect a complex interaction between an individual’s phenotype, i.e. morphological, behavioural, or physiological variability, and external constraints, such as resource abundance and availability or environmental heterogeneity^4^. In this context, long-distance migrants are a good model to explore the processes driving individual specialization. If individual specialization is maintained year-round, this would suggest that intrinsic constraints exist, such as cognitive processes, memory, physiological abilities, etc., which are part of individuals’ traits. On the contrary, specialization maintained only during a particular period of time would indicate a temporal adaptive response related to specific constraints or specific environmental conditions of that given period, such as energetic constraints related to breeding duties or disparate environmental predictability in breeding and non-breeding grounds. In this context, seabirds constitute an ideal model for studying the incidence and consequences of individual specialization, since many of these birds perform complex and long-distance migrations, exposing them to different environmental conditions in breeding and non-breeding grounds. Moreover, their long life span allows an exploration of consistency in different dimensions of the ecological niche over extended periods.

We aim to quantify the extent of individual specialization and its fitness implications in a long-distance migrant, the Cory’s shearwater (*Calonectris borealis*), using 74 adult individuals tracked for two to eight years both in breeding and non-breeding grounds and sampled in two breeding colonies of the Canary Islands. Specifically, we explore individual specialization in four dimensions of the ecological niche: non-breeding site fidelity, diet, habitat preference and daily activity (diurnal/nocturnal foraging) evaluated in breeding and non-breeding grounds. Non-breeding site fidelity was evaluated using global location sensors (GLS) devices, combined with stable isotope analyses (SIA) on feathers moulted in breeding and non-breeding grounds to infer seabirds’ diet. Habitat preference was inferred with sea surface temperature (SST) recorded by GLS devices. This variable can indicate habitat preferences within the upwellings, since many seabirds forage in upwelling systems, where the motion of deep cold waters towards the surface creates a contrasting gradient in temperature values from highly productive colder areas to oligotrophic warmer waters. Finally, daily activity was inferred using conductivity measured by GLS devices. Daily activity is a key component of seabirds’ foraging ecology, as the type of prey and its availability may change during the day and night^22^.

Cory’s shearwaters migrate to distinct areas throughout the Atlantic, and are subjected to different environmental conditions, types of prey and prey availability^20,23^. We thus hypothesize that individuals will show non-breeding site fidelity, as we expect it to be more beneficial to winter in a specific non-breeding area rather than adapting to different conditions every year. Indeed, non-breeding site fidelity has been previously found in Cory’s shearwaters from other colonies^20^. We also hypothesize that individual specialization will not be maintained year-round, as non-breeding areas are distantly located areas with different environmental conditions than those encountered at the breeding ground^23^. However, within breeding and non-breeding grounds, we expect individuals to be specialized in their diet, habitat preference and daily activity across years as a strategy to improve eficciency in resource explotation and to reduce intraspecific competition^4^. We also expect some individual trait preferences to have an impact on the reproductive success of individuals, as any biological trait with variability can potentially promote differences in fitness among individuals. Finally, we hypothesize that intermediate specialized individuals will show higher fitness, as highly specialized individuals will be less adapted to cope with environmental stochasticity and weakly specialized individuals will be less efficient in resource exploitation^4,24^.

## Results

### Non-breeding site fidelity

**Figure 1:**
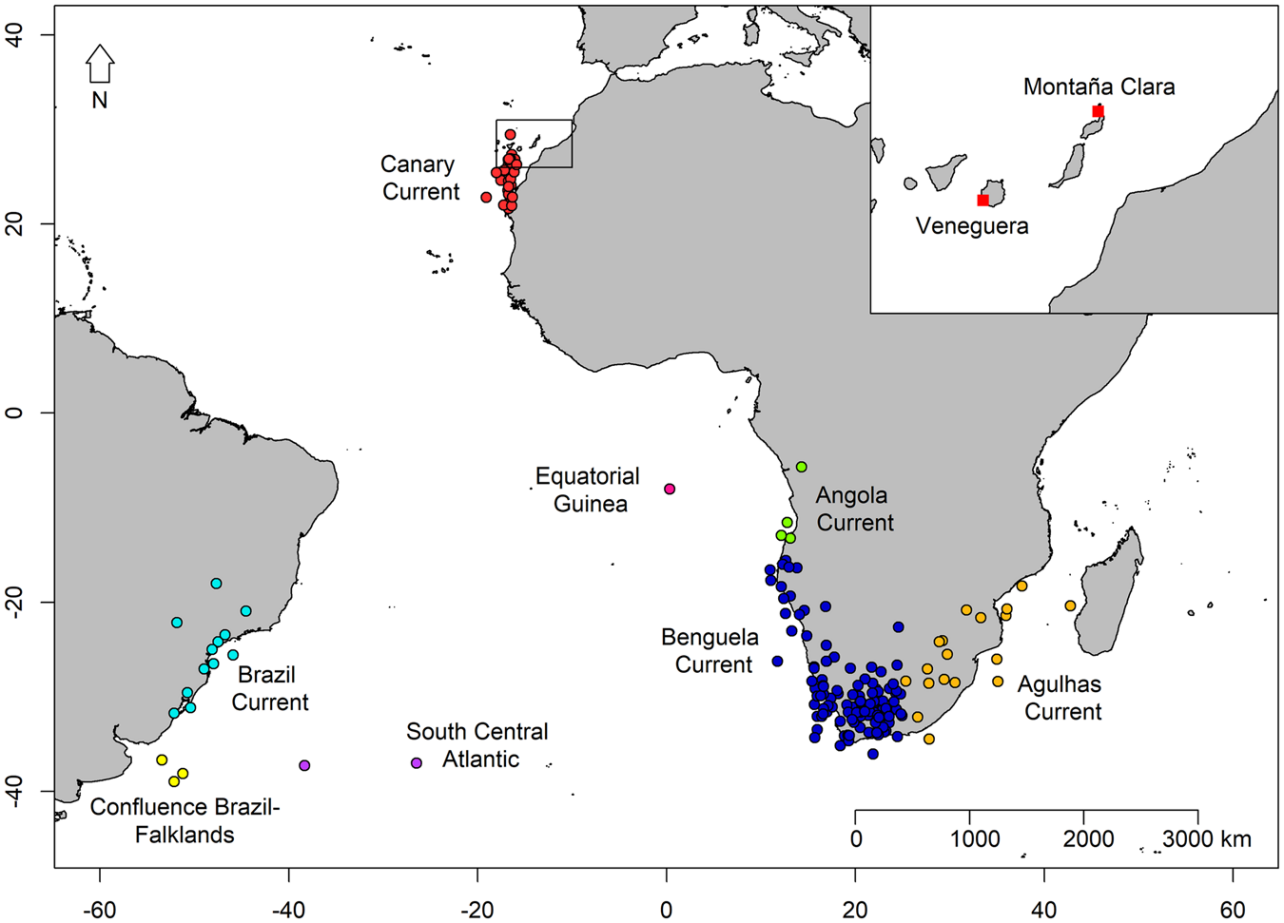
Non-breeding areas (points) and breeding colonies (red squares in the enlarged map, upper right-hand panel) of Cory’s shearwater from this study. Each point in non-breeding areas is the centroid of the non-breeding distribution of one individual in one year (N_ind_ = 59, N_trips_ = 176). Note that centroids over land are due to the intrinsic spatial error associated with light-level geolocation, since positions on land were not removed as they are subject to the same rate of error as positions at sea.

We obtained GLS data of 59 individuals: 32 tracked for two years, 10 for three years, seven for four years, six for five years and four for six years, comprising a total of 176 year-round trips. At a population level, we found that Cory’s shearwaters spent the non-breeding period in eight different areas (Fig. 1): Benguela Current (63.1% of migration events), Canary Current (14.2%), Agulhas Current (10.2%), Brazil Current (6.8%), Angola Current (2.3%), confluence of Brazil-Falklands/Malvinas Currents (1.7%), south central Atlantic (1.1%) and Equatorial Guinea Current (0.6%). We found that 21 of 59 individuals changed their areas at least once over the seven years of the study (i.e. 35.6%): 15 animals changed their non-breeding area once, five changed twice and one three times. Overall, we recorded 28 changes of non-breeding area from one year to the next in a total of 176 migration events (i.e. 15.9%). This corresponded to a Krippendorff’s alpha coefficient of 0.55 (95% CI: 0.37–0.69).

### Repeatability across years and year-round consistency

**Figure 2:**
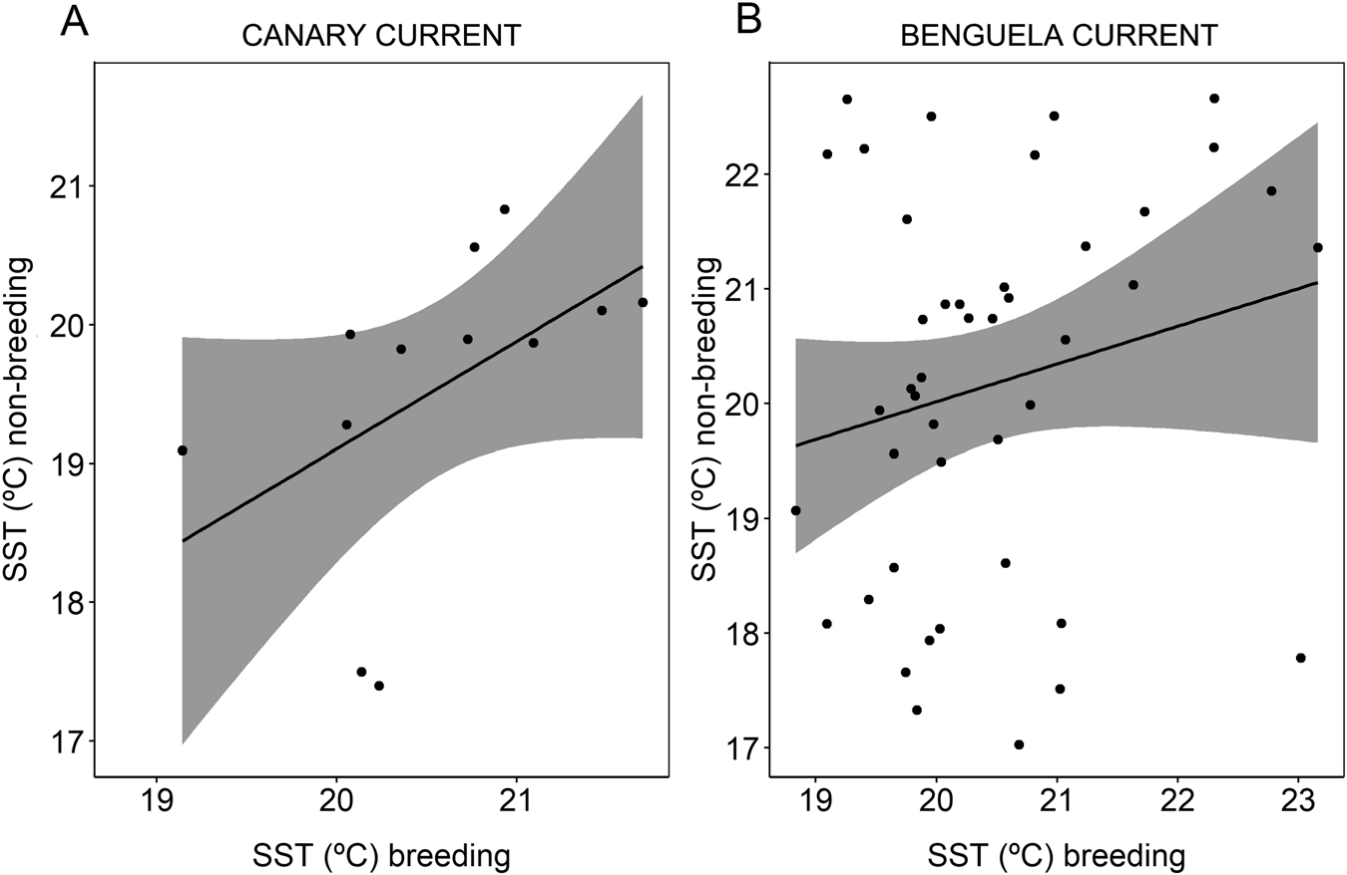
Relationship between sea surface temperature (SST) values during breeding and non-breeding periods of individuals spending the non-breeding period in the Canary Current (**A**; N = 12 values from six individuals, *χ*^2^_(1)_ = 8.9; p = 0.003, r^2^ = 0.50) and the Benguela Current (**B**; N = 44 values from 20 individuals, *χ*^2^_(1)_ = 0.02; p = 0.652, r^2^ = 0.04). Black lines correspond to the mean of the relationship and the grey shades are the associated 95% CI. Each point represents one individual per year.

**Figure 3:**
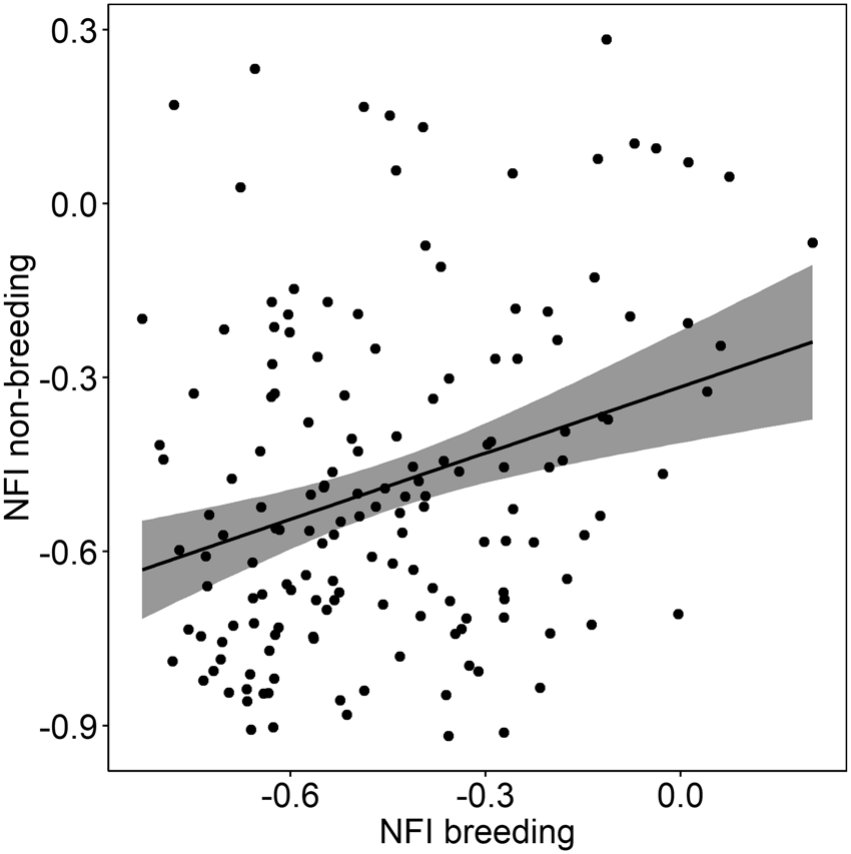
Relationships between night flight index (NFI) values during breeding and non-breeding periods (N = 158 from 53 individuals; *χ*^2^(1) = 13.00, p < 0.001, r^2^ = 0.15) only for birds spending the non-breeding period at the Benguela Current. The black line corresponds to the mean of the relationship and the grey shade is the associated 95% CI. Each point represents one individual per year.

We found repeatability to some extent in almost all proxies in all dimensions of the ecological niche, both in breeding and non-breeding grounds, namely isotopic diet (proxies δ^13^C and δ^15^N measured in two specific feathers: thirteenth secondary, hereafter S13, and the first innermost primary feather, hereafter P1), habitat preference (SST as a proxy) and daily activity (night flight index [NFI] as a proxy) (Table 1). Estimates of adjusted population repeatability values were statistically significant and thus trustworthy (95% confidence interval, CI, did not include 0), with the only exception being SST during breeding (Table 1). However, we did not find a correlation between breeding and non-breeding in δ^13^C, δ^15^N and SST values at an individual level, with the exception of SST in resident animals staying in the Canary Current year-round (Fig. 2). We found a significant positive correlation between breeding and non-breeding NFI values at an individual level (Table 1, Fig. 3). Moreover, in order to characterize habitat use in the context of available habitat, we looked at the range of SST in both breeding and in non-breeding grounds (for non-breeding only in Benguela due to the low sample size of the other areas). The range of SST values in the breeding ground (N = 101) was 6.0 °C (18.2–24.2 °C), whereas in the non-breeding ground (N = 44), the range of SST was 5.7 °C (17.0–22.7 °C).

**Table 1:** Summary of parameters for each dimension of the ecological niche.

Dimension of the ecological niche	Proxy	Period	N_total_ (N_ind._)	r^2^c	Adjusted population repeatability (*R*) across years (95% CI)	Year-round consistency (correlation breeding/non-breeding)
Diet	δ^13^C	Breeding (P1)	300 (74)	0.22	0.14 (0.04–0.28)	*χ*^2^_(1)_ = 1.2; p = 0.231
		Non-breeding (S13)	130 (43)	0.71	0.25 (0.06–0.52)	
	δ^15^N	Breeding (P1)	300 (74)	0.36	0.18 (0.08–0.32)	*χ*^2^_(1)_ = 0.3; p = 0.673
		Non-breeding (S13)	130 (43)	0.84	0.50 (0.34–0.70)	
Daily activity	NFI	Breeding	187 (61)	0.44	0.38 (0.23–0.56)	*χ*^2^_(1)_ = 13.0; **p < 0**.**001**
		Non-breeding	160 (54)	0.44	0.19 (0.03–0.41)	
Habitat	SST	Breeding	101 (36)	0.44	0.18 (0–0.44)	Benguela Current (N = 44): *χ*^2^_(1)_ = 0.02; p = 0.652 Canary Current (N = 12): *χ*^2^_(1)_ = 8.9; **p = 0**.**003**
		Non-breeding	64 (25)	0.72	0.45 (0.22-0.74)	

Significant breeding/non-breeding correlation are shown in bold. NFI is the night flight index, SST is sea surface temperature (°C) recorded by GLS devices and r^2^c is conditional r square of linear mixed models, i.e. the variability explained by the model including both fixed and random factors. Breeding/non-breeding correlations were performed only with animals spending the non-breeding season in the Benguela Current, except for SST which was also performed for animals wintering in the Canary Current. Note that adjusted population repeatability values obtained for all traits in both breeding and non-breeding grounds were statistically significant (i.e. 95% confidence interval CI > 0) and thus trustworthy, except for SST breeding. Note that sample size (N) is not the same in all cases because input data came from different sources of information (stable isotope analyses, immersion data from GLS devices and temperature data from GLS devices).

### Influence of individual trait preferences and degree of specialization on fitness

**Figure 4:**
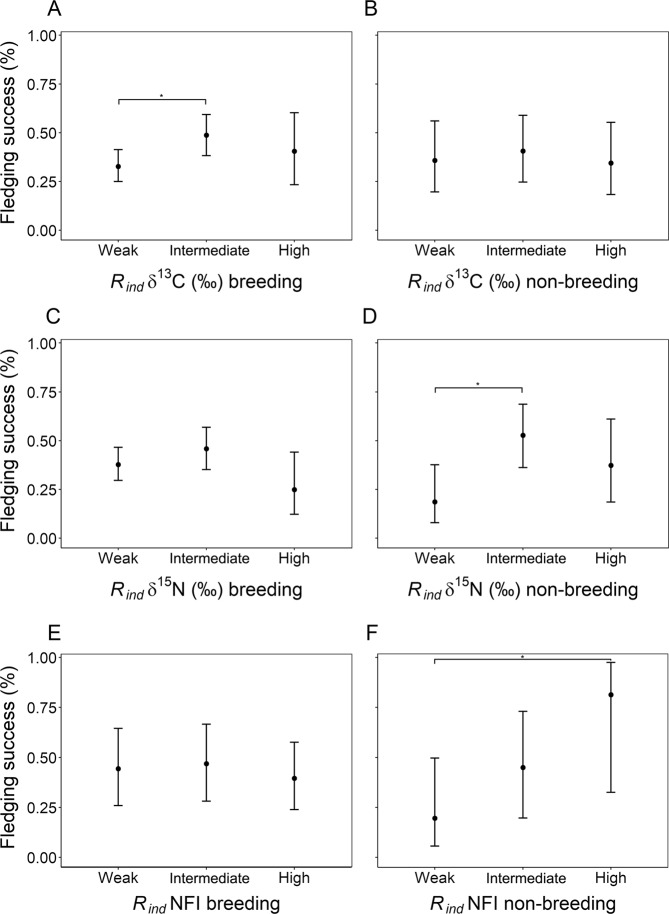
Probability of fledging success ± 95% CI depending on the degree of individual specialization estimated at the individual level (*R*_*ind*_) in δ^13^C (**A**,**B**), δ^15^N (**C**,**D**) and night flight index, NFI (**E,F**), during breeding (**A,C,E**) and non-breeding periods (**B,D,F**). Probability of fledging is calculated as the average fledging success (ranging from 0 to 1) for all years of each individual. Significant differences are shown with (*). Note than with δ^13^C and δ^15^N, intermediate specialized individuals tend to have higher mean fledging success than those weakly and highly specialized, although this difference is only significant between intermediate and weakly specialized in δ^13^C during breeding (Odds Ratio, OR = 1.96, 95% CI 1.02–3.75, N_intermediate_ = 22, N_weak_ = 37, p = 0.044) and in δ^15^N during non-breeding (OR = 4.89, 95% CI 1.72–13.94, N_intermediate_ = 10, N_weak_ = 10, p < 0.001). Note that with NFI during non-breeding, the higher the specialization, the higher the fledging success tends to be, although it is only significant between weakly and highly specialized individuals (OR = 17.93, 95% CI 2.73–117.95, N_intermediate_ = 5, N_high_ = 2, p < 0.001).

**Figure 5:**
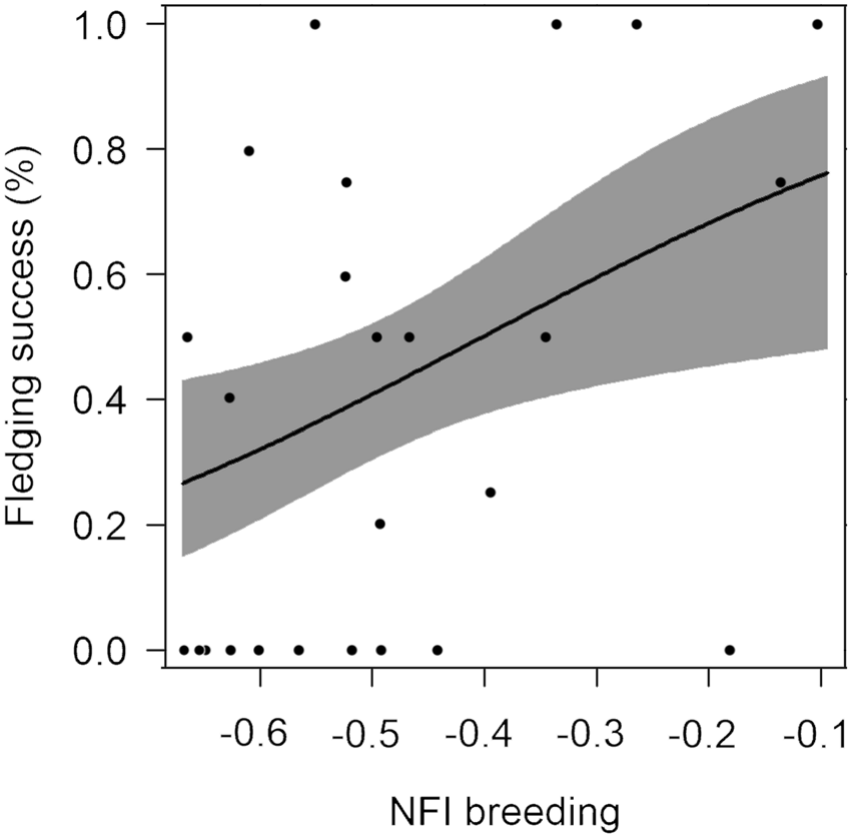
Probability of fledging success ± 95% CI in relation to individual mean values of night flight index (NFI) during breeding. Probability of fledging is the average fledging success (value ranging from 0 to 1) for all years of each individual. Note that during breeding, the higher the NFI values, the higher the fledging success (OR = 1.85, 95% CI 1.06–3.21, N = 27, p = 0.030).

We found different impacts of trait preferences and the degree of specialization on individual fitness, as inferred by fledging success. Regarding δ^13^C and δ^15^N, we found a general tendency of intermediate specialized individuals showing higher fledging success than those weakly or highly specialized (Fig. 4A–D). However, this pattern was only significant in two cases: intermediate specialized individuals showed higher fledging success than weakly specialized individuals in δ^13^C values during breeding (Odds Ratio, OR = 1.96, 95% CI 1.02–3.75, N_intermediate_ = 22, N_weak_ = 37, p = 0.044, Fig. 4A) and in δ^15^N during non-breeding (OR = 4.89, 95% CI 1.72–13.94, N_intermediate_ = 10, N_weak_ = 10, p < 0.001, Fig. 4D). Nevertheless, the mean level of δ^13^C and δ^15^N was not related to fledging success. In regards to NFI, during non-breeding there was a tendency for individuals with higher specialization in NFI to have higher fledging success, although the difference was only significant between weakly and highly specialized individuals (OR = 17.93, 95% CI 2.73–117.95, N_intermediate_ = 5, N_high_ = 2, p < 0.001, Fig. 4F). However, we did not find statistical evidence of an influence on the degree of NFI specialization during breeding (Fig. 4E). On the other hand, the more nocturnal that individuals were during breeding (higher NFI values), the higher their fledging success (OR = 1.85, 95% CI 1.06–3.21, N = 27, p = 0.030, Fig. 5), although we did not find any pattern during non-breeding. Regarding SST, we found that neither trait preferences nor the degree of individual specialization were related to fledging success.

## Discussion

In this study, we addressed inter-annual individual specialization in different aspects of the ecological niche and including the different conditions to which individuals are exposed, by studying several traits of the feeding ecology of Cory’s shearwaters in breeding and non-breeding grounds. In general, we found evidence of individual specialization in feeding strategies across years in both breeding and non-breeding grounds, although these individual strategies differed between grounds, suggesting the capacity of individuals to develop disparate strategies when environmental conditions differ. Moreover, the degree of individual dietary specialization seemed to influence fledging success, thus highlighting the importance of individual strategies on fitness.

Individual non-breeding site fidelity has been reported in many seabird species, such as skuas, puffins or albatrosses^6^. The first studies on Cory’s shearwaters suggested that individuals are plastic in their non-breeding destinations, with 36% of the individuals changing their non-breeding grounds from year to year, although this study was performed on only 13 individuals tracked for two years and one tracked for three years^23^. A more recent study with 51 individuals tracked for up to six years suggests that Cory’s shearwaters are less plastic than previously shown^20^. They found that 31% of individuals changed their non-breeding destination at least once during the study period, but that only 16% of individuals changed between consecutive migration events if we take into account all possible changes in all individuals and years^20^. In our study, we tracked 59 individuals for two to six years and found an overall strikingly similar result, with 36% of individuals changing non-breeding destinations and 16% of changes between consecutive migration events. Previous studies on shearwater species showed substantially higher non-breeding site fidelities than Cory’s shearwaters, including the streaked shearwaters (*Calonectris leucomelas*), in which just 2% of the individuals changed their grounds with 39 individuals tracked for two years and seven for three years^25^, and Scopoli’s shearwaters (*Calonectris diomedea*), which showed 0.46 repeatability in the distance travelled to non-breeding destinations with 10 individuals tracked for two years and two for three years^26^. These differences may emerge from a less complex migration system in these species, with both streaked and Scopoli’s shearwaters only having three different non-breeding destinations^25,27^, but may also be due to small sample sizes and fewer years considered in those studies. Overall, in this study Cory’s shearwaters used seven non-breeding areas across the Atlantic and Indian Oceans and some animals remained as residents in the Canary Current year-round, thus making its migration system more complex than the others. However, we should note that one of the non-breeding areas defined for streaked shearwaters was larger (3.4 million km^2^ approximately)^25^ than those defined in our study (up to 2.3 million km^2^ approximately). If this large non-breeding area of streaked shearwater was divided into smaller areas, as we did in our study, the percentage of change may have been greater.

Despite showing some flexibility in their non-breeding destination, Cory’s shearwaters showed specialization across years in the habitat exploited in non-breeding locations, as inferred from the high repeatability values (*R* = 0.45) in SST recorded by GLS. These values implies that individuals specialize in exploiting different foraging grounds within each non-breeding area, ranging from strictly neritic to the shelf slope waters or beyond.

Although shearwaters showed habitat specialization across years in non-breeding grounds, habitat specialization was not maintained all year-round. That is, the SST of the waters used during breeding and non-breeding were unrelated at the individual level, meaning that individuals changed their preferences when moving from breeding to non-breeding grounds. Indeed, there was no individual specialization in SST during breeding. Given that the range of SST was similar within breeding and non-breeding grounds, we can dismiss that these differences in specialization arose from a different range of available habitats in both areas. Ruling out differences in availability, different habitat specializations in breeding and non-breeding grounds could imply that such specializations are not driven by behavioural or physiological constrains, but are likely learned and fixed by experience early in life^28,29^. This process may occur independently in breeding and non-breeding areas, where environmental conditions are probably different, thus leading to different habitat use in each area. Indeed, only resident animals, spending the breeding and non-breeding periods in the same area (the Canary Current), showed a significant association between the SST used in the two periods, thus indicating changes in specialization between breeding and non-breeding period does not arise from the change in the period but from a change in the area.

Flexible preferences in environmental traits between breeding and non-breeding periods contrast with a year-round maintenance of individual strategies regarding daily activity, as observed by a significant positive correlation between breeding and non-breeding NFI values at an individual level. This implies, for instance, that birds that are more diurnal during breeding are also more diurnal during the non-breeding period. This suggests that daily behavioural specialization, once it is fixed, may have some returns regardless of the conditions in breeding and non-breeding grounds, such as an increased ability and efficiency in exploiting specific resources. However, this individual specialization maintained year-round may also imply some constraints on the foraging behaviour of individuals that could lead to sub-optimal foraging. Although NFI values showed that shearwaters are mainly diurnal animals, i.e. flight mainly occurs during daylight hours, there may be some individuals that are specialized in taking advantage of crepuscular hours to forage^30^. Diurnal individuals probably rely on pelagic fish^30^, whereas crepuscular birds probably depend on prey performing diel vertical migrations, as was previously found in other seabirds^22,31^. Indeed, the targeted prey of Cory’s shearwater includes prey that perform diel vertical migrations, such as Myctophidae species^32^. Individual specialization in daily activity has also been previously reported in seabirds. Similarly to the studied shearwaters, white-chinned petrel (*Procellaria aequinoctialis*) individuals specialized in their nocturnal activity^33^ and European shags (*Phalacrocorax aristotelis*) showed individual consistency in foraging either during morning or afternoon in daylight hours^34^.

Individual specialization in habitat and daily activity can lead to specialization in diet. Accordingly, we found that Cory’s shearwater individuals specialized in diet across years, as inferred by δ^13^C and, especially by δ^15^N values. δ^13^C values of a predator’ tissue are widely acknowledged to be good proxies of its food and habitat type^35^, while δ^15^N values indicate its trophic level^35^. It is known that Cory’s shearwaters mainly feed on epipelagic fish, although some birds can also rely on krill and cephalopods to some extent^32^. Since these prey show different δ^15^N values according to their trophic level, our results suggest that some individuals specialize on prey at low trophic levels, e.g. incorporating more krill, whereas others mainly feed on prey at mid-trophic levels, such as pelagic fish. Alternatively, higher δ^15^N values may also result from individuals scavenging on demersal fish discarded by trawlers, which typically has greater δ^15^N values than pelagic fish^36^. This may imply that some individuals specialize in naturally obtained pelagic prey, whereas others specialize on fishery discards to some extent. Indeed, fishery discard specialization has been reported in other seabirds, such as northern gannets (*Morus bassanus*)^37^, great skuas (*Catharacta skua*)^38^ or lesser black-backed gulls (*Larus fuscus*)^39^, although high flexibility regarding fishery waste utilization was also observed in black-browed albatross (*Thalassarche melanophrys*)^40^.

Although shearwaters showed diet specialization across years both, in breeding and non-breeding grounds, we did not find a correlation in the isotopic values between these two periods. This implies, for instance, that individuals with a high trophic level during breeding do not necessarily hold a high trophic level during non-breeding. Similar to the habitat inferred from SST, this result suggests that diet specialization may change between areas. Area-dependent dietary specialization may originate from changes in prey type between breeding and non-breeding areas. Indeed, target species of breeding Cory’s shearwaters are not necessarily found in non-breeding grounds. Namely, Atlantic horse mackerel (*Trachurus trachurus*) and European anchovy (*Engraulis encrasicolus)* are only present on the east coast of the Atlantic to northern Namibia, and are thus inaccessible to birds spending the non-breeding period in the west and central Atlantic and southern east African coast. Alternatively, different prey preferences depending on the area may originate from the observed differences in habitat preferences in each area, with neritic and oceanic habitats allocating different prey^41^. Accessibility to different prey can also explain the lack of correlation observed between breeding and non-breeding isotopic values of resident animals staying in the Canary Current year-round. Even when staying in the same area and habitat, prey accessibility can change due to seasonal movements, which occurs with sardines and horse mackerels performing seasonally latitudinal migrations in the Canary Current^42^.

The observed individual specialization in the different dimensions of the ecological niche may have consequences on individual fitness. We found some interesting tendencies regarding the implications of individual specialization on fitness, as indicated by fledging success. In the case of the diet, δ^13^C and δ^15^N values did not relate to fitness, but the degree of individual dietary specialization seems related to fledging success. Low and highly specialized individuals tended to exhibit lower fledging success than those medium specialized, although paired comparisons were only significant between intermediate and weakly specialized in δ^13^C during breeding and in δ^15^N during non-breeding. Higher fitness in intermediate individuals suggests stabilizing selection. This stabilizing selection, which crops the extremes and holds the intermediate phenotypes, is typical in stable environments with little variability^43^. Upwelling systems could be considered stable environments with high prey availability and spatiotemporal predictability. For instance, Benguela and Canary Current, were most individuals forage in the non-breeding and breeding period respectively^44^, are amongst the most important upwelling systems of the Atlantic^45^. In addition, shearwaters with a weak specialization in diet could be less efficient in resource exploitation than intermediate or highly specialized individuals, which may lead to lower breeding success^4,24,46^. Indeed, foraging specialization has been shown to increase breeding success in herring Gulls (*Larus argentatus*)^46^. Alternatively, a low breeding success in weakly specialized individuals may be related to the use of fishery discards. This resource is widely used by many seabird species, including shearwaters^47^, and it is also available in both, breeding and non-breeding grounds, of the studied shearwaters^48^. The exploitation of discards could be considered a specialized strategy, but the offered prey is variable in their isotopic values, which may lead to an apparently weakly specialized diet. Individuals exploiting discards may be less experienced and less efficient foragers and may also be those provisioning their chicks with suboptimal prey items from discards^49^. Highly specialized individuals showed similar or slightly lower breeding success than intermediate specialized ones, thus suggesting a limitation to positive effects of specialization, possibly related to a lower capability to cope with environmental stochasticity. Alternatively, the degree of specialization may just respond to different dietary strategies not necessarily related to fitness. Further research with higher sample sizes should ideally be undertaken to actually understand the influence of the degree of individual dietary specialization on the breeding success in long-lived species.

Contrarily, specialization in daily activity during non-breeding seemed to increase subsequent breeding success. However, taking into account the small sample size, with five weakly specialized, five intermediate specialized and two highly specialized individuals, these results should be interpreted with caution. Individual specialization in other aspects of the foraging strategies has been previously shown to be adaptive in other seabirds. For instance, successful breeders of black-browed albatross showed a substantially lower spatial niche than failed ones^50^, whereas repeatability in diving behaviour was related to foraging efficiency in great cormorants (*Phalacrocorax carbo*)^51^. Other studies with larger sample sizes are needed to understand the fitness implications of more nocturnal/diurnal behaviour on shearwaters.

In addition, we found more nocturnal animals during breeding to show higher concurrent breeding success. An increased fitness of more nocturnal animals could be related to the extensive fisheries operating on the Saharan coast of the Canary Current where shearwaters forage during breeding, which is clearly dominated by fisheries targeting epipelagic fish, such as sardines^52^. These type of fisheries are mainly active during crepuscule and night, when high concentrations of epipelagic fish are attracted to the surface by purse seine vessels using powerful lights. In this context, more nocturnal individuals may have large amount of their preferred prey available, thus increasing their body condition and ultimately their breeding performance.

## Conclusions

Overall, in this study we found that Cory’s shearwaters specialized in diet, daily activity and habitat from year to year. Additionally, daily activity behaviour was individually repeatable across seasons, independently of the areas visited by the individuals. Both results (i.e., repeatability between years and seasons) suggest that these animals may have a limited plasticity in their ecology and behaviour. Global changes, such as the forecasted increase of SST worldwide^53^, will not only alter the environmental conditions of the habitat itself, but may also alter abundance and distribution of prey^54^. In this scenario, low plasticity of predators could eventually compromise their ability to cope with environmental stochasticity and limit adaptive responses to global changes^55^.

Nevertheless, our study also showed Cory’s shearwaters are relatively flexible in their non-breeding destinations and are able to independently develop different habitat and dietary specializations under different environmental conditions. This suggests that individual specialization is not driven by individual constraints but can be acquired independently in different areas, suggesting the specialization process is more plastic than previously thought. Therefore, our study highlights the need to understand the underlying causes of individual specialization, in particular the need to distinguish between the specialization driven by permanent intrinsic traits of the individuals versus that promoted by temporal extrinsic factors, such as the stability or predictability of environmental conditions.

## Materials and Methods

### Fieldwork

Fieldwork was conducted from 2007 to 2015 in two colonies of Cory’s shearwaters (see Supplementary Material for basic information on the species): Veneguera (27°50′39.98″N, 15°47′19″E) and Montaña Clara (29°17′29″N, 13°31′57″E), both located in the Canary Islands. We deployed and re-deployed GLS loggers (global location sensor, models MK4, MK9, MK13, MK18-H and MK19 from British Antarctic Survey and MK3005 from Biotrack) on the same individuals whenever possible. GLS were recovered mainly after one year of deployment (but some of them after up to three years), thus reducing a potential bias of device id on anual measurements. The recovery rate was 93.7% (194 recoveries from 207 deployments). Of those recovered, 8.8% failed and had no light data during the non-breeding period to infer positions, thus resulting in 176 year-round tracks. GLS devices were attached to the leg of the animal with a PVC ring. We only included in this study birds tracked from two to eight years. At the time of recovering each GLS, we sampled S13 and P1 feathers, known to be moulted at the non-breeding and breeding grounds, respectively^56,57^. To link the specialization and strategies adopted by individuals to their fitness, we recorded fledging success by evaluating whether the chick from every monitored nest was alive around mid-October every year, when they are close to leaving the nest; this data was available for Veneguera but not for Montaña Clara.

### Assessing individual specialization

We analysed different dimensions of the ecological niche: non-breeding site fidelity, isotopic diet, daily activity behaviour and habitat preference. As explained below in detail, every dimension was analysed using a different source of information, which means that sample size and number of individuals will vary among them (see Table 1 for sample sizes).

We used the locations obtained from GLS devices to address non-breeding site fidelity. After processing raw GLS data, it is possible to infer two locations per day with an error of ±200 km^58^ based on timings of sunrise and sunset. We used TransEdit software to inspect light curves and to define dawn and dusk times daily. To filter erroneous locations, we removed the 30 days around the equinoxes and applied velocity filters by removing speed values higher than the 95^th^ percentile. Since shearwaters normally perform some stopovers and occasionally spend the non-breeding period in more than one area, we selected the main non-breeding area of each individual/year as the area where each animal spent the most days. For each individual and year, we calculated the kernel utilization distribution (UD) using positions within the main non-breeding area. To do so we used the function “kernelUD” from the package “adehabitatHR”^59^ and a smoothing parameter equivalent to ~2° to account for the spatial error in geolocation. Each centroid of the 5% kernel UD contour per individual and year was then assigned to one out of ten main non-breeding areas known to be used by the species, following the limits of the areas proposed by Militão *et al*. (2018)^60^.

To examine individual specialization in diet, we analysed stable isotope values of δ^13^C and δ^15^N for S13 and P1 feathers (see Supplementary Material for details on these analyses). To link trophic with the spatial information of the non-breeding period, each S13 feather sample was related to a non-breeding area inferred from GLS devices previous to the year of feather sampling, when that feather was moulted. P1 feathers were also linked to the previous year of sampling, as it was also the year of moult. To increase sample size during breeding, we included P1 feathers of animals not necessarily tracked with GLS devices.

Daily activity was assessed using the variation of behaviours (flying, resting) exhibited by individuals throughout the day and that are detected by GLS immersion data. GLS devices equipped with immersion sensor measure conductivity every three seconds and can provide information about two elemental states: wet, which means the animal was in contact with water and thus likely sitting on the sea surface, and dry, meaning the animal was flying and likely foraging. To address consistency in the daily activity, we calculated the NFI^23^. This index was calculated as the difference between the proportion of time spent in flight during darkness and during daylight, divided by the highest of these two values. Values of NFI range from −1, flight exclusively restricted to daylight, to 1, flight restricted to night. We defined day and night using the time of sunrise and sunset generated during the analysis of the light data from GLS devices. Per each individual and year, we calculated the mean of the daily values of NFI in the breeding and the main non-breeding area. Dry records in conductivity occur when birds are flying but also during nest attendance. To avoid misleading results, we selected a period of ~30 consecutive days just preceding the onset of post-nuptial migration to calculate NFI, since in this period animals were still at the breeding area but did not frequently visit the nest. We also selected a period of ~30 consecutive days in the middle of the period spent in the main non-breeding area, in order to avoid interferences of moonlight activity, which influence shearwaters at-sea behaviour^61^.

To understand habitat specialization, we used as a proxy the SST (°C) recorded by GLS devices, which is measured every 20 minutes after a minimum of 20 minutes of continuous contact with salt water. We calculated the mean SST per individual and year in breeding and non-breeding stages using the same 30-day window as with NFI. Cory’s shearwater are in contact with salt water when drafting and diving, and maximum diving depth is 6–7 meters^62^. Therefore, GLS-recorded temperatures can be considered as SST.

### Statistical analyses

All statistical analyses were carried out using R 3.4.3^63^. Prior to statistical analyses, we checked for normality of δ^13^C, δ^15^N, NFI and SST values using Kolmogorov-Smirnov tests and Q-Q plots. As no severe deviations from normality were found, we used parametric tests throughout. Significant levels in all analyses were set to 0.05. To disregard the possibility that GLS id inflated repeatability values of GLS measurements (SST and NFI), we first included GLS id in the models and in all cases was not significant.

We used Krippendorff’s alpha coefficient to estimate how repeatable individuals were in the selection of a specific non-breeding area, i.e. non-breeding site fidelity. This index can be calculated with several years of data and multiple individuals, allowing missing data for some individuals in some years and also taking into account the number of possible non-breeding areas to which individuals can go. This index ranges from 0, meaning the same individual constantly changes its non-breeding area, to 1, meaning the same individual always selects the same non-breeding area. We calculated it using “krip.alpha” function in the “irr” R package^64^ and performed bootstrapping with 10,000 iterations to obtain the 95% CI of the estimate^65^.

Repeatability is a commonly used statistic in behavioural studies to address individual specialization^16^. For the traits δ^13^C, δ^15^N, NFI and SST, we addressed individual specialization by calculating repeatability (*R*), Eq. (1):

1 $$R=\frac{{s}_{A}^{2}}{{s}_{W}^{2}+{s}_{A}^{2}}$$

where *R* is repeatability, s_A_^2^ is the variance among individuals and s_W_^2^ is the variance within individuals, with s_W_^2^ + s_A_^2^ being the total variation in the sampled population. The value of *R* ranges from 0 to 1, 0 meaning the trait has no repeatability and the majority of individuals do not show specialization (i.e. population is mainly composed of generalists) and 1 meaning that trait is highly repeatable and individual specialization is at a maximum in a given population (i.e. most individuals of the population do show specialization). Note that in order to be repeatable, a trait must be consistent within individuals but different among individuals^66^. Among and within individual variances can be directly estimated from residuals of Linear Mixed-Effects Models, LMM^15,66^. Moreover, LMM allow the control of possible effects of confounding factors by including them as fixed factors or covariates, thus providing an adjusted repeatability^15,66^. We estimated adjusted repeatability (*R* hereafter) through LMM using the “rpt” function in the “rptR” package, which returns the mean value of *R* in the population and its 95% confidence interval (CI) calculated by parametric bootstrapping^67^. A 95% CI that does not include 0 means the value of *R* is statistically significant. We calculated *R* for the traits δ^13^C, δ^15^N, NFI and SST, separately for breeding and non-breeding. Each LMM included “year” (the year of moult in the case of isotopic values from feathers), “sex” and “non-breeding area” (the latter only in non-breeding models) as fixed factors and “individual” as a random factor. The factor “colony” (either Veneguera or Montaña Clara) was also added for all models except δ^13^C and δ^15^N during breeding, since we do not have P1 from Montaña Clara. We ran 10,000 iterations for each model to obtain the 95% CI. We included year as a fixed factor to keep the possible effects of annual variability out of the residual variance of the models. Animals were sexed either with molecular sexing (68% of individuals) or using discriminant functions (32%) from fieldwork bill measurements. For those models related to non-breeding, we only considered non-breeding areas with a sample size larger than five individuals/year.

To explore the consistency of traits throughout the annual cycle, we performed LMM with the same structure explained previously but also including as a covariate the parameters calculated during breeding, in order to explain the same trait during the following non-breeding season. As an example, NFI during breeding was used as covariate to explain NFI during non-breeding. We used the “lmer” function in the “lme4” package^68^ to run the models. Significance of the non-breeding covariate was estimated using the “mixed” function in the “afex”^69^ package with the likelihood ratio test.

To address the effect of individual strategies on fitness, we explored the influence of individual trait preferences and degree of specialization on the probability of fledging success. Considering each individual and year for which the nest was monitored at the time of fledging (mid-October), we proceeded as follows. First, we calculated the average fledging success (value ranging from 0 to 1) for all years of each individual. We later calculated the average value of each trait (δ^13^C, δ^15^N, NFI and SST) for all years of each individual. From the previous LMM performed to calculate *R*, we extracted the individual repeatability value (*R*_*ind*_ hereafter) associated with each trait, following previous approaches^51^. We considered individuals to be weakly, intermediate or highly specialized according to their *R*_*ind*_ value. To classify them objectively, we used the k-means algorithm for clustering *R*_*ind*_ values into three groups (note that mean and size of each group varies depending on the range of *R*_*ind*_ values in each trait, see summary in Table 1 of Supplementary Material). Finally, we fitted logistic regression models (Generalised Linear Models, GLM, with logit-link function and binomial error distribution) to model the probability of fledging success, including the number of years fledging was recorded as a weight into the models. We included as predictors the *R*_*ind*_ group (fixed factor) and the mean value of its associated trait (covariate). The weakly specialized group was set as a reference level. To ensure model suitability, we tested uniformity of residuals and accounted for overdispersion for every GLM using functions provided by the “DHARMa” package^70^. To evaluate the effect of predictors, both the p-value and 95% CI of coefficient estimates were calculated. Pairwise comparisons among levels were calculated based on estimated marginal means and adjusted using post-hoc Tukey correction^71^. Regarding mean values of traits, in the cases of δ^13^C and δ^15^N, we used the fledging success following the year of moult in the case of non-breeding variables and of the year of moult in the case of breeding variables. Similarly, for NFI and SST we used the fledging success following the non-breeding period in the case of non-breeding variables and of the concurrent breeding attempt in the case of breeding variables. Regarding non-breeding, we only performed GLM with individuals spending the non-breeding period in the Benguela system, because it was the only area with N > 5.

### Ethics

All experiments were performed in accordance with relevant guidelines and regulations and all protocols were approved by Gobierno de Canarias (permits: 84/2007, 2011/0795, 2015/1170).

## Supplementary information

Supplementary information

## Data Availability

Data will be available at Universitat de Barcelona archive.
