# Supplementary material for: Year-round individual specialization in the feeding ecology of a long-lived seabird
Source: Sci Rep. 2019 Aug 14;9:11812. doi: 10.1038/s41598-019-48214-0 (PMC6694139; doi:10.1038/s41598-019-48214-0)
Supplement: Supplementary file 1 — Supplementary information [file 41598_2019_48214_MOESM1_ESM.pdf]

Year-round individual specialization in the feeding ecology of a long-lived seabird

Laura Zango, José Manuel Reyes-González, Teresa Militão, Zuzana Zajková, Eduardo Álvarez-Alonso, Raül Ramos, Jacob González-Solís

Supplementary information

Study model

Cory's shearwater is a long lived pelagic seabird from Procellariidae family that breeds in Azores, Madeira, Salvagems and Canary archipelagos, as well as in occidental Mediterranean in Terreros and Chafarinas. It is a long distance migrant spending the non-breeding period in west and south African coast, from Angola to South Africa, as well as in Uruguay and Brazil coast, and with some individuals also wintering in the Guine-Equatorial current and others oceanic waters in the central Atlantic. This species usually keeps well away from land, except during the breeding period (del Hoyo et al. 2014). It is globally classified as Least Concern (IUCN 2017). The species feeds mostly on fish, squid and crustaceans, although there are evidences of interactions with trawlers to feed on fishery discards (Traversi & Vooren, 2010).

Stable isotope analyses

P1 and S13 feathers were washed in a 0.25 M sodium hydroxide solution (NaOH), rinsed twice with distilled water and dried in an oven at 40°C. Whole feathers were powdered in a cryogenic impact grinder (Freeser/mill Spex Certiprep 6750; Spex) operating at liquid nitrogen temperature. We placed 0.25-0.30 mg of each sample in a 3.3x5 mm tin cup and used this subsample to obtain $\delta^{13}C$ and $\delta^{15}N$ values in a continuous-flow isotope-ratio mass spectrometry (CF-IRMS) at Serveis Cientifico-Tècnics of Universitat de Barcelona. Isotope ratios are expressed conventionally as δ values in part per thousand (‰) according to the following equation:

$$\delta X = [(Rsample/Rstandard) - 1]$$

Where X (‰) is $\delta^{13}C$ or $\delta^{15}N$ and R are the corresponding ratio $^{13}C/^{12}C$ or $^{15}N/^{14}N$ related to the standard values. Standard for $\delta^{13}C$ is Vienna Pee Dee Belemnite (VPDB) and for $\delta^{15}N$ is atmospheric nitrogen (air). Internal laboratory standards (Acetanilide, IAEA CH6, USGS 42) indicated average measurement errors of 0.2‰ for both $\delta^{13}C$ and $\delta^{15}N$ values.

Table 1 Supplementary Material. Summary of individual repeatability values (R_{ind}) for each group from k-means algorithm, namely weakly, intermediate and highly specialized. NFI is the night flight index and SST is sea surface temperature in ºC.

**Trait**	**Group R_{ind}**	**Breeding**				**Non-breeding**			
		N	Mean R_{ind}	Min R_{ind}	Max R_{ind}	N	Mean R_{ind}	Min R_{ind}	Max R_{ind}
**$\delta^{13}C$**	Weak	37	0.15	0.04	0.27	9	0.15	0.08	0.25
	Intermediate	22	0.43	0.32	0.62	9	0.35	0.30	0.45
	High	7	0.84	0.79	0.95	7	0.74	0.56	0.95
**$\delta^{15}N$**	Weak	36	0.17	0.03	0.29	10	0.16	0.04	0.28
	Intermediate	21	0.43	0.31	0.54	10	0.44	0.33	0.53
	High	9	0.70	0.59	0.90	5	0.78	0.62	0.89
**NFI**	Weak	7	0.49	0.27	0.55	5	0.36	0.21	0.43
	Intermediate	9	0.69	0.61	0.77	5	0.58	0.49	0.65
	High	11	0.88	0.81	0.93	2	0.76	0.70	0.81
**SST**	Weak	7	0.19	0.11	0.31	9	0.41	0.28	0.50
	Intermediate	5	0.47	0.38	0.59	5	0.66	0.65	0.66
	High	4	0.81	0.67	0.97	2	0.95	0.84	0.99
